# Cluster headache with ptosis responsive to intranasal lidocaine application: a case report

**DOI:** 10.1186/1752-1947-6-64

**Published:** 2012-02-15

**Authors:** Berker Bakbak, Sansal Gedik, Bengu Ekinci Koktekir, Mehmet Okka

**Affiliations:** 1 Department of Ophthalmology, Selcuk University Selcuklu Medical Faculty, Konya, Turkey; 2 Department of Ophthalmology, Selcuk University Meram Medical Faculty, Konya, Turkey

## Abstract

**Introduction:**

The application of lidocaine to the nasal mucosal area corresponding to the sphenopalatine fossa has been shown to be effective at extinguishing pain attacks in patients with a cluster headache. In this report, the effectiveness of local administration of lidocaine on cluster headache attacks as a symptomatic treatment of this disorder is discussed.

**Cases presentation:**

A 22-year-old Turkish man presented with a five-year history of severe, repeated, unilateral periorbital pain and headache, diagnosed as a typical cluster headache. He suffered from rhinorrhea, lacrimation and ptosis during headaches. He had tried several unsuccessful daily medications. We applied a cotton tip with lidocaine hydrochloride into his left nostril for 10 minutes. The ptosis responded to the treatment and the intensity of his headache decreased.

**Conclusion:**

Intranasal lidocaine is a useful treatment for the acute management of a cluster headache. Intranasal lidocaine blocks the neural transmission of the sphenopalatine ganglion, which contributes to the trigeminal nerve as well as containing both parasympathetic and sympathetic fibers.

## Introduction

Cluster headache (CH) is defined as a paroxysmal, strictly unilateral and very severe headache [1]. It is seen very rarely with a prevalence of less than 0.1% [2]. Ptosis, miosis, lacrimation, conjunctival injection, rhinorrhea and nasal congestion are autonomic symptoms, which usually accompany retro-orbital pain. CH can be categorized into episodic and chronic forms. Various treatment modalities have been tried in both the prevention and treatment of CH [3-6]. The intranasal application of 10% lidocaine to the nasal mucosa corresponding to the sphenopalatine fossa has proved effective [3,6]. However, the mechanism of lidocaine in treating the headache is unknown. Here, we report a male patient suffering from CH and severe ptosis that immediately resolved with intranasal lidocaine application and discuss the possible mechanism of lidocaine in treating CH.

## Case presentation

A 22-year-old Turkish man presented with a five-year history of intermittent daily headache centered on the left retro-orbital and orbital side. The pain was unilateral with a side shift only within the same bout. He experienced four to twenty attacks a week from the beginning of the bout, which resulted in severe social agitation. The attacks started abruptly and usually peaked within five minutes, without any aura or precipitating factors, and lasted 30 minutes to 120 minutes. He suffered from rhinorrhea, lacrimation and ptosis during the headaches, without any noted nausea, vomiting or photophobia. He had previously used several daily medications unsuccessfully, such as verapamil 160 mg thrice daily, naproxen 500 mg thrice daily, ibuprofen 600 mg thrice daily, dexketoprofen trometamol 25 mg twice daily, indomethacin 25 mg thrice daily, loratadine 5 mg daily and prednisolone 60 mg daily. Both general and neurological examinations between attacks and hematological-biochemical screenings were normal. He had neither significant past medical history nor family history of headache.

On the day of a severe headache, an ophthalmological examination of our patient revealed lacrimation, conjunctival injection and ptosis without miosis. We measured both his pupils as 3.5 mm, with normal pupillary reactions to both light and near stimulation. As attacks occurred without significant periods of remission, we diagnosed our patient with chronic CH. We applied a cotton tip with 2 mL of lidocaine hydrochloride and epinephrine (Jetocaine, 20 mg lidocaine/0.025 mg epinephrine) into the left nostril for 10 minutes. The ptosis responded to the treatment and the intensity of his headache decreased. The magnetic resonance (MR) images of his brain and orbit and MR angiography of his brain and carotid artery were within normal limits. In 12 months of follow-up, he had six to ten attacks a week accompanied by autonomic symptoms, which resolved with intranasal lidocaine application.

## Discussion

CH is a type of primary headache characterized by severe, unilateral trigeminal pain with cranial parasympathetic autonomic symptoms involving oculocephalic functions [3]. The estimated prevalence is less than one in 1,000 in the general population and the disease affects men with a sex ratio between two point five and seven point five to one [2]. The diagnostic criteria of the International Headache Society allow for the distinction of two main CH subtypes, namely an episodic form and a chronic form [1,4]. In the episodic form, attacks occur daily for some weeks followed by a period of remission. In the chronic form, attacks occur without significant periods of remission.

The unilateral autonomic symptoms such as ptosis, miosis, lacrimation, conjunctival injection, rhinorrhea and nasal congestion are lateralized to the pain during the pain attack, and indicate parasympathetic hyperactivity and sympathetic impairment [4,6].

CH attacks are extremely painful and have a very rapid onset and a very short duration. Acute therapy aims to abort individual attacks. Treatment should therefore be able to resolve or significantly reduce pain and accompanying autonomic symptoms. Acute therapy for CH includes oxygen inhalation and administration of ergots, triptans, analgesics and intranasal local anesthetic agents [4-7]. However, currently there is no specific therapy to relieve the symptoms.

Practitioners have used lidocaine as an acute treatment option for many types of headache by suppository, intramuscular, intravenous and nasal routes. Lidocaine 4% application in the sphenopalatine fossa may offer the fastest relief of any known agent [3,8,9]. The sphenopalatine ganglion (SPG) resides just posterior to and immediately above the posterior tip of the middle turbinate, beneath the nasal mucosa at a depth of 1 mm to 9 mm. Intranasal lidocaine is administered with the patient supine, with the tip of the nose pointed vertically, and the head turned slightly toward the side of the block. A cotton-tipped applicator saturated with 4% lidocaine is inserted intranasally and applied to the lateral posterior wall of the nasal cavity. The application of lidocaine to the area corresponding to the sphenopalatine fossa has been shown to be effective at extinguishing pain attacks in patients with CH. Robbins [7] reported the clinical features and results of the treatment of 30 patients with CH who had tried 4% lidocaine solution as a nasal spray to abort the attacks. Of these 30 patients, 27% of the patients reported moderate relief, 27% obtained mild relief and 46% found no relief from the lidocaine.

Costa *et al*. [3] conducted a placebo-controlled study in nine CH patients on the effect of a 1 mL solution of 10% lidocaine applied during nitroglycerine-precipitated CH attacks, applying a cotton swab intranasally on both sides in the area of the sphenopalatine fossa under anterior rhinoscopy. In all treated patients, the pain disappeared on average within 37 minutes of lidocaine application. It has been demonstrated that nasal congestion, rhinorrhea, lacrimation and photophobia generally disappear with pain, while conjunctival injection, miosis and ptosis are resolved later [6]. In our case, following intranasal lidocaine application, pain ceased first and then ptosis resolved, as described in the literature. We encountered no complications in our case and, to date, there is no reported toxicity in the literature.

The mechanism for lidocaine in treating CH is unknown. Lidocaine provides its anesthetic effect as a sodium pump inhibitor. It is hypothesized that lidocaine provides local anesthesia, blocking neural transmission of the SPG, which may be important in CH pathophysiology [9]. The SPG is a complex region, including sensory fibers that contribute to the trigeminal nerve, as well as both parasympathetic and sympathetic fibers. Therefore, intranasal lidocaine may produce both sensory and parasympathetic nerve blockade. However, the mechanisms by which these blockages occur are not clear.

## Conclusion

CH attacks need a symptomatic treatment that has a rapid onset of action. Intranasal lidocaine is a useful treatment for the acute management of CH, because of its rapid onset of action and comfortable self-administration. Patients can learn this technique themselves in order to relieve acute attacks while conventional therapy is being optimized.

## Consent

Written informed consent was obtained from the patient for publication of this case report and any accompanying images. A copy of the written consent is available for review by the Editor-in-Chief of this journal

## Competing interests

The authors declare that they have no competing interests.

## Authors' contributions

SG, BEK and MO analyzed and interpreted the patient data. BB was a major contributor in writing the manuscript. All authors read and approved the final manuscript.
